# Inhibition of BET proteins modulates amyloid-beta accumulation and cognitive performance in middle-aged mice prenatally exposed to maternal immune activation

**DOI:** 10.3389/fnmol.2025.1619583

**Published:** 2025-07-14

**Authors:** Marta Matuszewska, Anna Wilkaniec, Magdalena Gąssowska-Dobrowolska, Magdalena Cieślik, Gabriela Olech-Kochańczyk, Ewelina Pałasz, Elżbieta Gawinek, Marcin Strawski, Grzegorz A. Czapski

**Affiliations:** ^1^Department of Cellular Signalling, Mossakowski Medical Research Institute Polish Academy of Sciences, Warsaw, Poland; ^2^Faculty of Chemistry, University of Warsaw, Warsaw, Poland

**Keywords:** prenatal exposure delayed effects, inflammation, beta amyloid, bromodomain containing proteins, hippocampus

## Abstract

**Introduction:**

Given the complex etiological basis of Alzheimer’s disease (AD), it is reasonable to hypothesize that neuronal dysfunction and death result from the interplay of numerous factors, both genetic and environmental. Accumulating evidence implicates the immune system and inflammation as key components of the pathomechanism of AD. In the present study, we analyzed the effect of maternal immune activation (MIA) on AD-related pathological changes in middle-aged 12-month-old offspring mice. Additionally, we investigated whether the inhibition of bromodomain and extraterminal domain (BET) proteins, which are readers of the histone acetylation code, could influence these changes.

**Methods:**

In our study, we administered a viral mimetic, polyinosinic-polycytidylic acid (PIC), on gestation day 17 to induce MIA in wild-type C57BL/6J mice. The BET protein inhibitor, OTX-015 (Birabresib), was administered orally to 12-month-old male offspring for 14 days. Subsequently, behavioral, genetic, and immunochemical analyses were conducted.

**Results:**

Our results demonstrated several MIA-evoked molecular alterations in the brains of middle-aged offspring. We observed an increase in *App* gene expression (qPCR) and amyloid-*β* (Aβ) levels (ELISA), while the levels and phosphorylation of Tau protein remained unchanged (WB). The mRNA levels of selected microglial markers were also elevated in the MIA group. Treatment with OTX-015 improved memory, as observed in the novel object recognition test, and reduced Aβ levels, but did not alter the expression of inflammatory genes or amyloidogenesis-related genes.

**Discussion:**

Our findings suggest that inhibition of BET proteins may effectively attenuate neuropathological alterations in the aged brain.

## 1 Introduction

Alzheimer’s disease (AD) is a heterogeneous disorder characterized by a multifaceted pathomechanism and an extended prodromal phase. The complex interplay between genetic, epigenetic, and environmental factors, encompassing both risk and protective elements, contributes to variability in disease dynamics, clinical manifestations, amyloid-*β* (Aβ) conformation, and Tau protein distribution (Ferreira et al., 2020; Ferrari and Sorbi, 2021). Consequently, a singular and simplistic explanation of the pathomechanism of AD is unlikely to exist. Multiple hypotheses have been advanced to explain the underlying mechanisms of AD, including dysfunction in cholinergic and glutamatergic neurotransmission, the role of infections and inflammatory processes, amyloid and/or Tau propagation and accumulation, lymphatic system involvement, neurovascular changes, calcium dyshomeostasis, metal ion imbalance, and the mitochondrial dysfunction (Liu et al., 2019; Yokoyama et al., 2022).

Among these, the role of inflammatory processes has gained particular attention due to mounting evidence from epidemiological, genetic, and experimental studies. The immune system-related release of mediators of inflammation with potentially neurotoxic properties was proposed as a detrimental factor over two decades ago (McGeer and McGeer, 1998). Activation of glial cells around plaques and tangles occurs linearly with progression of the AD (Serrano-Pozo et al., 2011). Furthermore, genome-wide association studies (GWAS) have identified many genetic risk factors associated with AD, the majority of which are linked to immune system functionality (Bertram and Tanzi, 2019). Also, epidemiological studies confirmed that prolonged treatment using non-steroidal anti-inflammatory drugs (NSAIDs) reduced the risk of developing AD (t'Veld et al., 2001; Côté et al., 2012). However, the clinical trials involving NSAIDs demonstrated the complexity of the pathomechanism of AD, because the effects of NSAIDs changed during the disease, being protective or noxious (Breitner et al., 2011; Leoutsakos et al., 2012). Finally, population-based studies demonstrated that regular viral or bacterial infections may accelerate the progression of the disease and enhance the risk of developing AD (Bu et al., 2015; Douros et al., 2021; Sun J. et al., 2022). For example, an association was found between periodontal bacteria and faster cognitive decline in AD (Borsa et al., 2021).

This focus on immune dysregulation aligns with emerging discussions about shared mechanisms between neurodevelopmental disorders (NDDs) and neurodegenerative diseases like AD (Boots et al., 2023; Wiegersma et al., 2023; Siguier et al., 2024). It is suggested that factors contributing to the pathomechanisms of NDDs may elevate the risk of developing dementia through mechanisms related to a lessened cognitive reserve, genetic factors, or physiopathological overlaps (Siguier et al., 2024). Maternal infections affect the functional architecture of the brain and are associated with neurodevelopmental and neuropsychiatric problems, including autism spectrum disorders, schizophrenia, or ADHD in the progeny (Kim et al., 2024). Inflammatory signaling in the mother’s body during pregnancy can disturb well-orchestrated and vulnerable processes of the central nervous system’s development, including cells’ proliferation, differentiation, migration, synaptic formation and pruning, and the establishment of neuronal circuits (Knuesel et al., 2014; Cieślik et al., 2020a; Cieślik et al., 2020b). Data suggest that the underlying cause of neurodevelopmental disturbances is nonspecific and not directly dependent on the pathogen itself (Woods et al., 2023). Instead, it is associated with the processes activated during the response of the maternal immune system to infection.

The identification of shared pathways in NDDs and AD prompted us to investigate whether maternal immune activation (MIA), a known risk factor for NDDs, could induce AD-like alterations in the brains of aging offspring. To solve this puzzle, we used a viral mimetic, polyinosinic-polycytidylic acid (PIC), to induce MIA in pregnant wild-type mice. Then, we analyzed 12-month-old male offspring. Since MIA is believed to evoke long-lasting epigenetic alterations in offspring (Woods et al., 2021; Kleeman et al., 2022), we investigated the potential involvement of bromodomain and extraterminal domain (BET) proteins. BET proteins, the readers of chromatin acetylation code, collaborate with transcription factors in governing the expression of genes (Liu et al., 2021). Data directly linking long-lasting MIA-induced epigenetic changes specifically to BET protein-regulated genes are limited. However, alterations in the global histone acetylation profile and changes in histone deacetylase levels in the hippocampus have been observed in 8–12-week-old offspring mice prenatally exposed to PIC-induced MIA (Reisinger et al., 2016). Additionally, PIC-induced MIA in rats has been shown to evoke changes in the global histone acetylation profile in the prefrontal cortex of 60-day-old offspring, including increased H3 and H4 histone acetylation at the promoter region of *Rela*, a gene regulated by BET proteins (Wang et al., 2018; Su et al., 2022). Therefore, in our study, OTX-015, an inhibitor of BET proteins, was administered orally (voluntary intake) to 12-month-old offspring males for 14 days. Our results demonstrated that inhibiting BET proteins attenuated MIA- induced elevation of brain Aβ level and improved memory.

## 2 Materials and methods

### 2.1 Materials

High molecular weight polyinosinic-polycytidylic acid (HMW PIC) was from InvivoGen (San Diego, CA, United States). (6S)-4-(4-chlorophenyl)-N-(4-hydroxyphenyl)-2,3,9-trimethyl-6H-thieno[3,2-f][1,2,4]triazolo[4,3-a][1,4]diazepine-6-acetamide (OTX-015) was from Biorbyt Ltd. (Cambridge, UK). BCA Protein Assay Kit, TRI-reagent, reagents for reverse transcription (High-Capacity cDNA Reverse Transcription Kit with RNase Inhibitor), reagents for quantitative PCR (Taqman Assays and TaqMan Fast Advanced Master Mix), ELISA kits for mouse Aβ_1-40_ and Aβ_1-42_ were obtained from Thermo Fisher Scientific, Inc. (Waltham, MA, United States). Kits for quantitative detection of mouse bromodomain-containing proteins 2, 3, and 4 (BRD2, BRD3, and BRD4) using the enzyme-linked immunosorbent assay ELISA Kit were obtained from Abbexa Ltd. (Cambridge, UK). Clarity Western ECL Substrate was purchased from Bio-Rad Laboratories (Hercules, CA, United States). Peanut butter (smooth, 100% peanuts) was from Sante sp. z o.o. (Warsaw, Poland). Protease inhibitors cocktail Complete was purchased from Roche Diagnostics (Mannheim, Germany). DNase I, dithiothreitol (DTT), anhydrous dimethyl sulfoxide (DMSO), and all other reagents were obtained from Sigma-Aldrich (St. Louis, MO, United States). Primary antibodies: rabbit anti-GAPDH Ab, mouse anti-Tau Ab, and rabbit anti-pTau(Ser199/202) Ab were from Sigma-Aldrich, mouse anti-pTau(Ser396) Ab, rabbit anti-pTau(Ser404) Ab, rabbit anti-pTau(Ser416) Ab, and rabbit anti-Iba1 Ab (for WB) were from Cell Signalling Technology (Danvers, MA, United States), goat anti-GFAP Ab (for WB), goat anti-Iba1 Ab (for IHC), and rabbit anti-GFAP Ab (for IHC) were from Abcam (Cambridge, UK). Secondary HRP-conjugated antibodies: anti-mouse IgG (GE Healthcare Bio-Sciences AB, Uppsala, Sweden), anti-rabbit IgG (Sigma-Aldrich), and anti-goat IgG (Santa Cruz Biotechnology, Dallas, TX, United States). Fluorochrome-conjugated secondary antibodies: Alexa Fluor 488 donkey anti-rabbit IgG and Alexa Fluor 594 donkey anti-goat IgG were from Thermo Fisher Scientific, Inc. Vectashild Vibrance Antifade Mounting Medium with DAPI was from Vector Laboratories, Inc. (Newark, CA, United States).

### 2.2 Animals

The experiments were carried out on C57BL/6J mice, supplied by the Animal House of Mossakowski Medical Research Institute, Polish Academy of Sciences (Warsaw, Poland), which operates breeding of small rodents with the specific-pathogen-free (SPF) standard. The animals were maintained under controlled temperature (22°C ± 10%) and humidity (55% ± 10%) conditions on a 12 h light/dark cycle. All experiments carried out on animals were approved by the Local Ethics Committee for Animal Experiments in Warsaw (reference number WAW2/052/2021) and carried out following the ARRIVE guidelines and the EU Directive 2010/63/EU regarding animal experiments. Every effort was made to minimize the number of animals used and to reduce the risk of animals’ pain and distress. For the whole experiment, we used 16 pregnant females, resulting in 48 male offspring. The use of male offspring mitigated the confounding effects of the estrous cycle in females on the experimental data.

### 2.3 Experimental design—maternal immune activation

The mice gestations were realized by housing an adult male and a female overnight. The following day, female mice were separated, and the pregnant ones were identified and randomly assigned to the experimental group. At gestation day 17 (GD17), MIA was evoked in 8 pregnant females by intraperitoneal (i.p.) administration of HMW PIC (20 mg/kg b.w.) (Krstic et al., 2012; Mueller et al., 2019). Eight pregnant females from the non-MIA group received i.p. administration of an analogous volume of vehicle (sterile 0.9% NaCl). All dams were allowed to give birth and nurture offspring under normal conditions. Dams from MIA group gave birth to 27 male offspring, and dams from non-MIA group gave birth to 21 male offspring. On postnatal day (PND) 22 to 23, male pups were separated and housed in groups of 3 or 4 in open polycarbonate cages in an enriched environment. For the current project, all 21 offspring males from the MIA group, and randomly selected 21 males from non-MIA group were utilized. The remaining offspring were allocated to other projects.

#### 2.3.1 Characterization of PIC—atomic force microscopy

Multimode 8 Nanoscope atomic force microscope (AFM, Bruker, Unites States) was used to image the surfaces on V1 grade mica substrate (NanoAndMore GmbH, Germany). Silicon cantilevers, HQ: NSC19/No Al type with a spring constant of ca. 0.5 Nm^−1^ (Mikromasch, Bulgaria) were applied for imaging in PeakForce Tapping^™^ microscopy mode. The image presented in this work is height type images. The examination of surfaces for artefacts by AFM, and the reproducibility, was performed in the common way, i.e., by changing the AFM cantilever and moving the sample in the X or Y direction, or by varying the scanning angle and scan rate. Polyinosinic-polycytidylic acid samples were prepared by applying a drop of 10 μL medium, which was previously diluted in 10 mM MgCl_2_ solution in a 1:100 v/v ratio, on a freshly cleaved mica. Divalent magnesium is commonly used as a linking agent between negatively charged mica surfaces and DNA/RNA chains (Lyubchenko, 2011). After incubation for 10 min, the sample was rinsed with deionised water (GENIE U 12 TOC + TR, RephiLe Bioscience Ltd.) and dried under a gentle stream of argon.

#### 2.3.2 Characterization of PIC—agarose gel electrophoresis

Electrophoresis was performed on a 1% agarose gel containing ethidium bromide to assess the quality and size of HMW-PIC. Low molecular weight (LMW) PIC was also loaded on the gel for comparison. A 100–1,000 bp DNA ladder was used as a size marker. After electrophoresis, the gel was visualized under UV light to detect the nucleic acids.

#### 2.3.3 Drug administration

**Figure 1:**
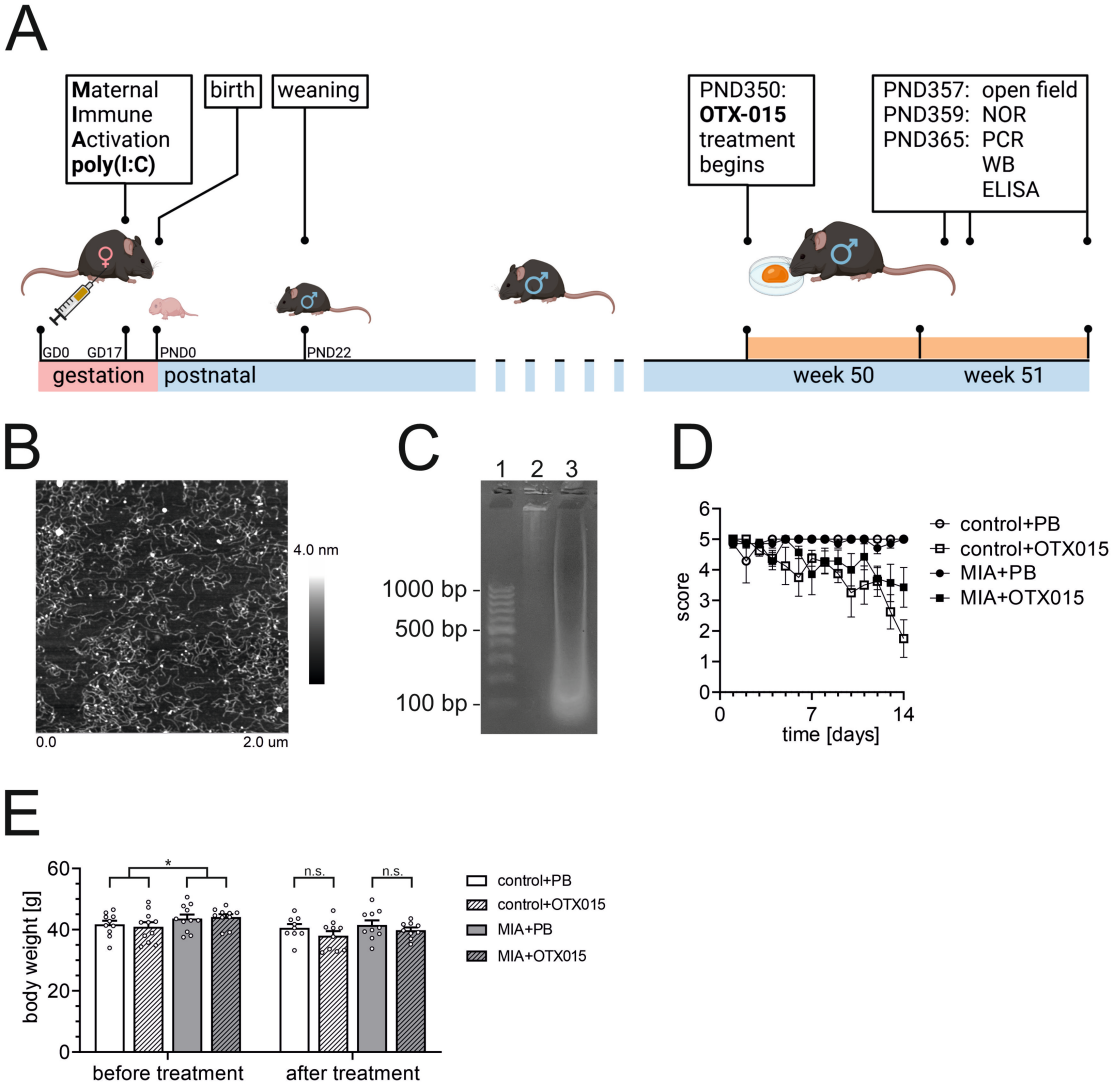
The design of the study. **(A)** The experimental setup (picture created with biorender.com). PIC (20 mg/kg b.w.) was injected intraperitoneally at gestation day 17 to female mice. An inhibitor of BET proteins, OTX-015 (100 mg/kg b.w. daily), was administered orally to 12-month-old offspring males for 14 days (weeks 50–51), then animals were euthanized, and the brain tissue was analyzed; **(B)** the typical Atomic Force Microscopy picture of polyinosinic-polycytidylic acid (PIC); **(C)** the typical picture of electrophoretic analysis of high molecular weight (HMW) PIC (lane 2) and low molecular weight (LMW) PIC (lane 3) on agarose gel, size markers range is 100–1,000 bp (lane 1); **(D)** propensity of mice to voluntary drug intake during 14 days of treatment, the score calculation was based on time of voluntary butter/drug intake: 5 – 0–15 min, 4 – 15–30 min, 3 – 30–45 min, 2 – 45–60 min, 1 – above 60 min, 0 – no intake; PB – peanut butter, MIA-maternal immune activation. **(E)** The effect of MIA and OTX-015 on the body weight of 12-month-old offspring animals. The presented data are means ± SEM. *N* = 7–8 **(D)** and 9–11 **(E)**. Each data point (○) represents an individual animal. * *p* < 0.05. Statistical analyses were conducted using either the Student’s t-test (to compare groups before OTX-015 treatment) or one-way ANOVA (to compare all groups), as appropriate based on data distribution.

To minimize stress, pain, and morbidity, oral self-administration of the drug by voluntary intake by 12-month-old animals was performed, using 100% natural smooth peanut butter (PB) as a vehicle (Hocking et al., 2018; Warren et al., 2021). The drug/peanut butter was given individually to each mouse on a polystyrene Petri dish (diameter 30 mm) in a separate cage. Drug self-administration of each mouse was performed in the same treatment cage throughout the experiment, and each mouse was in its own separate cage. The treatment cages were of the same type as home cages. To familiarize mice with the taste of PB, all mice were trained by giving 50 μL of PB per mouse five times during one week before the experiment. The OTX-015 (or vehicle in respective groups) was given at the end of the lights-off phase of the light–dark cycle. After consuming the drug/PB, every mouse was transferred directly to the home cage. Typically, 15–30 min was enough to consume the whole mixture. Self-administration of OTX-015/PB was performed daily at a dose of 100 mg/kg b.w. for 14 days (Figure 1A). Animals were randomly allocated to experimental groups: 10 in control group, 11 in MIA group, 10 in MIA + OTX-015 group, and 11 in OTX-015 group.

### 2.4 Behavioral analysis

Behavioral analysis was performed on all 42 12-month-old animals. After 7 days of self-administration of the drug, behavioral analysis was started as described previously (Czapski et al., 2021). The open field (OF) test may measure general locomotor activity, novel environment exploration, and anxiety-related behavior during 5 min. Animals were individually placed in the corner of the open field chamber (grey box, 55 cm × 55 cm × 30 cm), and the total distance and exploration of the central zone and the border zone were analyzed. The number of episodes of grooming, rearing, climbing, and defecation was counted by the blinded operator. After two days of rest, a novel object recognition (NOR) test was performed. This test exploits the natural tendency of rodents to explore novel objects to test non-spatial memory. One day before testing, mice were submitted to a habituation session; they were allowed to freely explore the test chamber (dark grey box, 30 cm × 20 cm × 30 cm) for 5 min. The experimental session consisted of two trials. In the first trial (T1), two identical objects (O1) were placed in the chamber. During the second trial (T2), one object, O1, was replaced with the alternative object, O2. For testing the objects, we used a set of plastic bricks and a cell culture bottle. The objects presented during sessions were free of olfactory traits, and their positions in the chamber were randomized to eliminate the spatial bias in the task. At the beginning of each trial, mice were placed at the center of the box, with their heads oriented in the opposite direction to the object. The duration of T1 and T2 was 5 min. T2 started 120 min after T1. The basic measurement was the total time spent by mice exploring objects during T1 and T2 trials. Exploration of an object was defined as follows: directing the nose at a distance of 2 cm to the object and/or touching it with the nose. Climbing time was excluded from the analysis. The index of discrimination (ID) was calculated for each animal in the T2 trial and expressed as a ratio: time spent exploring the novel object/added time of exploring novel and known objects. All behavioral tests were performed in the morning, from 8 a.m. to noon.

### 2.5 Sample collection

After 14 days of voluntary drug administration, animals were deeply anesthetized (with ketamine/xylazine for immunocytochemistry or isoflurane for other analyses) and sacrificed. Brain samples for biochemical analysis (30 animals) were collected, cooled in ice-cold PBS, and the whole hippocampus was immediately dissected with the chilled scalpel on the cold dissection tray. Dissected tissue was immediately snap frozen, left and right side, separately. From each animal, the left and right sides were used for RNA and protein extraction in a random manner. For IHC staining (12 animals), mice were perfused with 4% paraformaldehyde before decapitation and brain removal. All samples were frozen and stored at −80°C upon analysis.

### 2.6 Western immunoblotting

The immunoreactivity of proteins was analyzed as described previously (Gąssowska-Dobrowolska et al., 2021). Tissue samples were homogenized in RIPA buffer, and protein concentration was determined using the BCA method, with bovine serum albumin as a standard. Samples were mixed with Laemmli buffer and heated at 95°C for 5 min. After SDS-PAGE, proteins were transferred to a nitrocellulose membrane in standard conditions and then used for immunochemical analysis, followed by chemiluminescent detection using Clarity Western ECL Substrate. Densitometric analysis was performed using TotalLab4 software (NonLinear Dynamics Ltd., Newcastle upon Tyne, UK) using glyceraldehyde 3-phosphate dehydrogenase (GAPDH) level for data normalization.

### 2.7 Enzyme-linked immunosorbent assay

Enzyme-linked immunosorbent assay (ELISA) kits were used strictly according to the manufacturer’s protocols. Prepared samples were used fresh to prevent protein degradation and denaturation. Each sample was analyzed in duplicate.

Shortly, for analysis of the level of Aβ_1-40_ and Aβ_1-42_, brain tissue was homogenized in a cold buffer (5 M guanidine-HCl, 50 mM Tris, pH 8.0). Then, samples were left on a laboratory rocker at room temperature for 4 h. The sample was then diluted tenfold with cold PBS with a protease inhibitor cocktail and centrifuged at 16,000 × g for 20 min at 4°C. The supernatant was transferred to clean microcentrifuge tubes for further analysis according to the manufacturer’s protocol.

For the analysis of the level of BET proteins, brain tissue was homogenized in ice-cold PBS (pH = 7.2) using a syringe, and then, due to the tendency of chromatin-associated BET proteins to form insoluble complexes, a sonication was performed to increase their solubility (Lambert et al., 2009). This was performed on the samples for 30 s (40% pulse, 40% power) using a Model 150 V/T Ultrasonic Homogenizer from Biologics Inc. (Manassas, VA, United States). Then, the homogenates were centrifuged at 10,000 × g for 5 min, and the supernatant was collected. After all incubation steps were completed, optical density was measured at 450 nm using a Multiskan GO Microplate Spectrophotometer (Thermo Fisher Scientific, Inc.). The concentration of the tested compound was calculated using a standard curve and normalized to the total protein level in sample. Protein concentration was quantified using the BCA assay with bovine serum albumin (BSA) as the standard.

### 2.8 Quantitative real-time polymerase chain reaction (qRT-PCR)

RNA was isolated by using a TRI-reagent according to the manufacturer’s protocol. The concentration and quality of RNA were measured using a Nanodrop 2000 spectrophotometer (Thermo Fisher Scientific, Inc.). Digestion of potential DNA contamination was performed by using DNase I, according to the manufacturer’s protocol (Sigma-Aldrich). Reverse transcription was performed using a High-Capacity cDNA Reverse Transcription Kit with RNase Inhibitor according to the manufacturer’s protocol (Thermo Fisher Scientific, Inc.). Quantitative PCR was performed on an ABI 7500 Real-Time PCR System using TaqMan Fast Advanced Master Mix according to the manufacturer’s instructions (Thermo Fisher Scientific, Inc.). To increase the validity and reproducibility of qPCR analysis, the ΔΔCt calculation was extended by replacing the Ct of a single reference gene with an averaged Ct-value from three reference genes (*Gusb*, *Hprt*, *Rn18s*) (Riedel et al., 2014). The level of mRNA for selected genes was analyzed using commercially available TaqMan Gene Expression Assays: *Abca1* (Mm00442646_m1), *Adam10* (Mm00545742_m1), *Aph1b* (Mm00781167_m1), *App* (Mm01344172_m1), *Arg1* (Mm00475988_m1), *Bace1* (Mm00478664_m1), *Brd2* (Mm01271171_g1), *Brd3* (Mm01326697_m1), *Brd4* (Mm01350417_m1), *Gusb* (Mm01197698_m1), *Hprt* (Mm00446968_m1), *Il1b* (Mm00434228_m1), *Il6* (Mm00446190_m1), *Mme* (Mm01285049_m1), *Ncstn* (Mm00452010_m1), *Nos2* (Mm00440502_m1), *Psen1* (Mm00501184_m1), *Psen2* (Mm00448405_m1), *Rn18s* (Mm03928990_m1), *Tnf* (Mm00443258_m1).

### 2.9 Immunofluorescence staining

Mice were anesthetized with a mixture of ketamine and xylazine (100 mg/kg b.w. and 10 mg/kg b.w., respectively) and perfused through the ascending aorta initially with 0.9% NaCl in 0.1 M PBS, pH 7.4, and then with 4% paraformaldehyde. Brains were removed and post-fixed for 3 h at 4°C in the same fixative solution. Following post-fixation, brains were cryoprotected overnight in 20% sucrose solution in 0.1 M PBS, frozen on dry ice, and stored at −80°C. Coronal sections (40 μm thickness) were washed 3 times with 0.1 M PBS + 0.3% Triton X-100 for 5 min and incubated in a blocking solution (5% normal donkey serum (NDS) in 0.1 M PBS + 0.3% Triton X-100) for 1 h at room temperature (RT). The incubation with primary antibodies was performed in 1% BSA, 0.3% Triton X-100, and 0.1 M PBS for 1 h at RT and overnight at 4°C. The next day, the sections were washed with 0.1 M PBS (3 × 5 min), incubated in the dark with fluorescently labeled secondary antibodies in 1% BSA, 0.3% Triton X-100, and 0.1 M PBS for 1 h at RT, and washed with 0.1 M PBS (3 × 5 min). The sections were then mounted onto glass slides, air dried, and coverslipped with Antifade Mounting Medium with DAPI. Negative controls were performed using the same procedure, omitting the primary antibodies. Immunofluorescence studies were conducted in the Laboratory of Advanced Microscopy Techniques MMRI PAS using a confocal laser-scanning microscope, Zeiss LSM 780/ELYRA PS.1. (Carl Zeiss Meditec AG, Jena, Germany) platform equipped with the ZEN 2012 software, lasers (488 or 561 nm), and 405 nm diode lamp. Images were captured using a Zeiss PLN-Apo 40× /0.95 DIC III objective and further magnified by a 2 × digital zoom, resulting in an effective magnification of 80×. Z-stack acquisitions were performed from set first to set last planes, typically consisting of approximately 20 slices. Each optical slice had a thickness of 1.2 μm and was acquired at 1 μm intervals. For Sholl analysis, Z-stack images were converted from XYZ to XY dimensions using maximum intensity projection (MIP). Images were optimized for color, brightness, and contrast for best clarity. Multiple-channel images were overlaid using ZEN light software.

The morphometric analysis of microglia in the CA1 field of the hippocampus was performed according to the method described previously (Babiec et al., 2023). For every tested animal, up to nine microglial cells were randomly selected from the pyramidal cell layer, up to nine cells from the stratum radiatum, and up to nine cells from the lacunosum-moleculare, and their values were averaged to give three independent values. The extensions of individual cells were traced using the NeuronJ plugin for ImageJ Fiji software (Schindelin et al., 2012). The surface area of the cell body (soma), the number of primary and secondary extensions, and the total length of processes belonging to each cell were analyzed. The branches of individual cells were determined using the Sholl method (Sholl, 1953). Each cell was analyzed by selecting the center of its soma. Then, the number of intersections at circles of increasing diameter from the center was counted using the SNT plugin for ImageJ Fiji (Arshadi et al., 2021).

The semiquantitative analysis of microglia and astroglia density within the CA1, CA2/3 and DG region of the hippocampus was performed by manually counting Iba1- or GFAP-positive cells in six randomly selected fields (200 × 200 μm) per region per animal. Fields were selected with three from the left and three from the right hemisphere. The procedure was performed by a blinded operator and followed the method described previously (Babiec et al., 2023).

### 2.10 Statistical analysis

The statistical analysis of data was performed by using GraphPad Prism version 8.3.0 (GraphPad Software, San Diego, CA, United States). The distribution of data was analyzed using the Shapiro–Wilk test. The results were expressed as mean values ± SEM. Data were analyzed using either a Student’s t-test or one-way analysis of variance (ANOVA) with Bonferroni post hoc test for multiple comparisons correction for data with Gaussian distribution, or Mann–Whitney test or Kruskal-Wallis test with Dunn post hoc test for multiple comparisons correction for data with non-Gaussian distribution. *p*-values < 0.05 were considered significant. The *N* number refers to independent samples (biological replicates). To reduce the risk of litter effects, animals from at least 3 litters in each experimental group (random selection) were tested.

## 3 Results

In our study, we used a mouse model of MIA induced by intraperitoneal injection of PIC at gestation day 17 (GD17) (Figure 1). The murine MIA model based on PIC administration is well-characterized (Hameete et al., 2021). Therefore, to be consistent with the 3Rs principle (replacement, reduction, and refinement), we decided not to confirm activation of the immune system in pregnant mice. MIA did not affect gestation outcome in mice; the number of animals in the MIA group was unchanged, as compared to the non-MIA group (data not shown). Also, MIA did not influence the average time of voluntary intake of peanut butter (PB) or OTX-015. However, the average time of voluntary intake of OTX-015 was longer than that of PB (data not shown). For detailed analysis, we implemented a score-based approach, which demonstrated that the propensity of animals to voluntary intake of PB did not change during the 14-day-long treatment. Still, the propensity of animals to voluntary intake of OTX-015 decreased during the treatment (Figure 1D). We also observed that directly before the OTX-015 treatment, the body weight of animals in the MIA group was ca. 6% higher than that of the control group (Figure 1E). During the 14-day-long treatment with PB or OTX-015, the body weight did not increase (Figure 1E).

**Figure 2:**
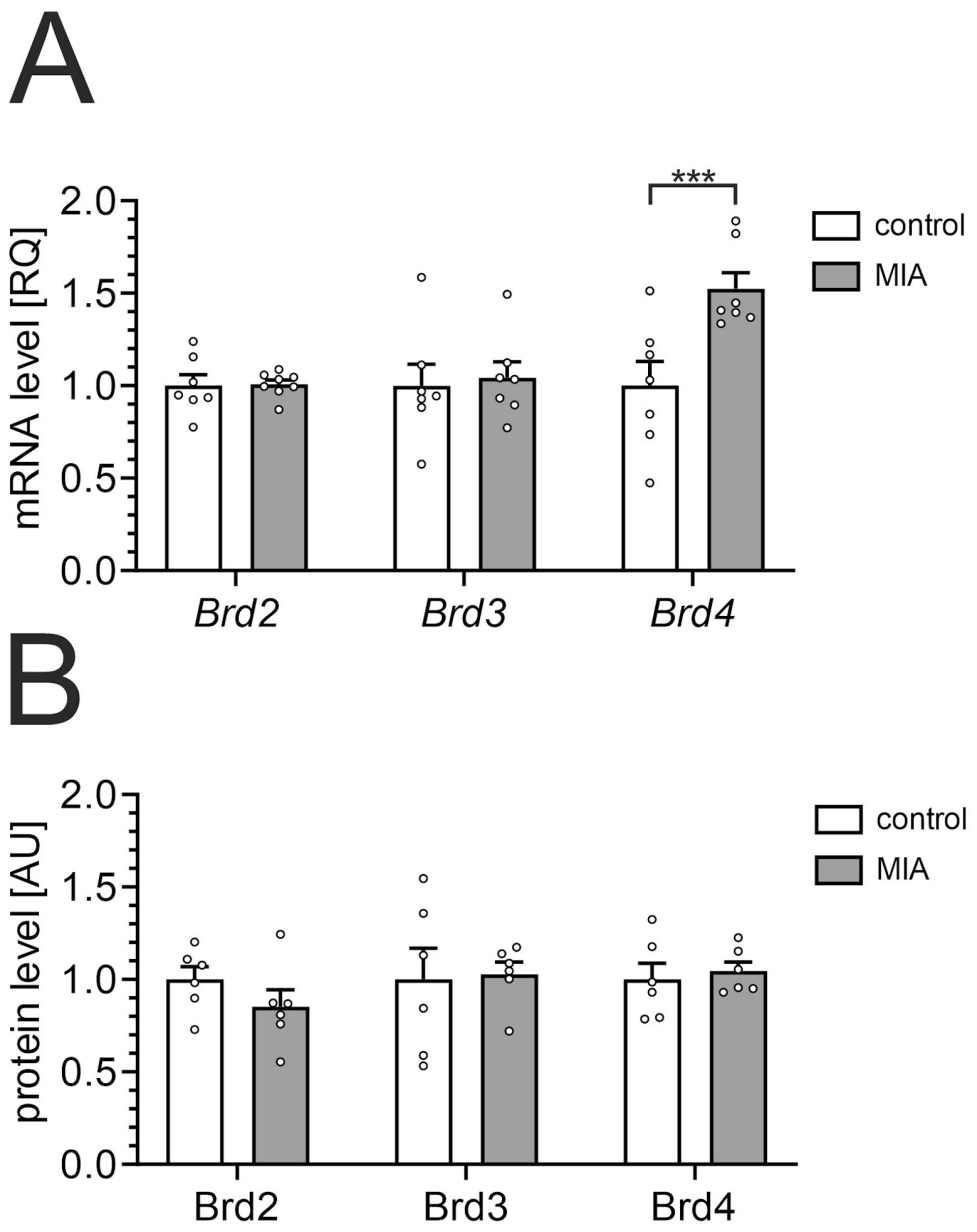
The effect of MIA on the expression of BET proteins in the hippocampus. PIC (20 mg/kg b.w.) was injected intraperitoneally at gestation day 17 to female mice. Twelve-month-old offspring male mice were decapitated, and the brain tissue was collected. **(A)** The levels of mRNA for *Brd2*, *Brd3*, and *Brd4* genes in the hippocampus were measured by using qPCR and calculated by the ΔΔCt method. An averaged Ct-value from three various reference genes (*Gusb*, *Hprt*, *Rn18s*) was used for calculation. **(B)** The levels of Brd2, Brd3, and Brd4 proteins in the hippocampus were determined by using ELISA assays and normalized to the total protein level. The presented data are means ± SEM. *** *p* < 0.001, compared with the control group. *N* = 7–8 **(A)** and 6 **(B)**. Each data point (○) represents an individual animal. Statistical analyses were conducted using two-way ANOVA followed by the Bonferroni post hoc test, selected based on data distribution.

The effect of MIA on the level of BET proteins in the hippocampus of 12-month-old animals was analyzed using qPCR and ELISA methods. In our study, we focused on the hippocampus, a crucial brain region for memory and learning. The hippocampus is also one of the first brain regions to exhibit pathological alterations in AD (Ball et al., 1985; Jaroudi et al., 2017). Furthermore, it is a structure highly sensitive to early life stress and inflammatory insults (Dantzer et al., 2008; Czapski et al., 2010; Delpech et al., 2016). Our data showed that MIA significantly increased the mRNA level for the *Brd4* gene, but mRNA levels of *Brd2* and *Brd3* were not changed (Figure 2A). However, BET protein levels, measured by ELISA assays, did not show a significant change in the hippocampus of MIA-exposed animals (Figure 2B). Under control conditions, Brd2, Brd3, and Brd4 showed comparable abundance, measured at 1.04 ± 0.07, 1.65 ± 0.28, and 0.26 ± 0.02 pg/μg of protein, respectively.

**Figure 3:**
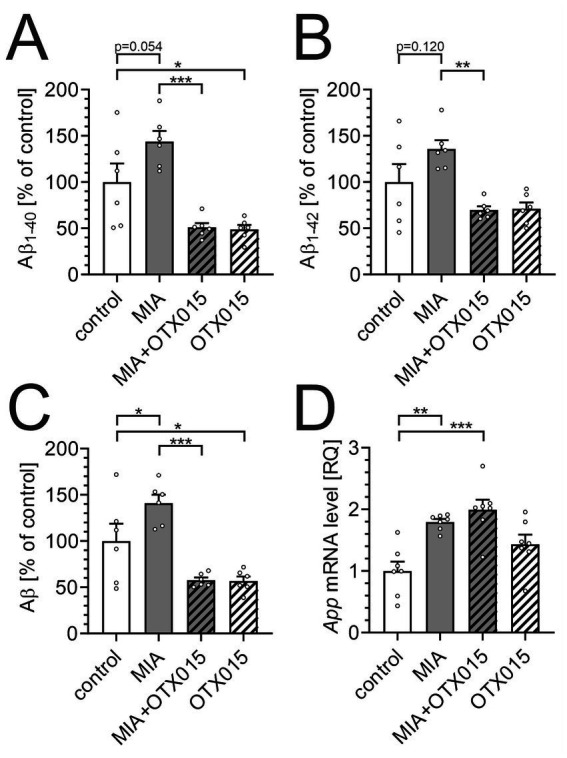
The effect of MIA and OTX-015 on Aβ level. PIC (20 mg/kg b.w.) was injected intraperitoneally at gestation day 17 to female mice. An inhibitor of BET proteins, OTX-015 (100 mg/kg b.w. daily), was administered orally to 12-month-old offspring males for 14 days (weeks 50–51), then animals were decapitated, and the brain tissue was collected. The levels of Aβ_1-40_ in the hippocampus **(A)** and the level of Aβ_1-42_ **(B)** were determined by using ELISA assays and normalized to the total protein level. The total level of Aβ **(C)** in the hippocampus was calculated by summing levels of Aβ_1-40_ and Aβ_1-42_. The level of mRNA for *App* gene in the hippocampus **(D)** was measured by using qPCR and calculated by the ΔΔCt method. An averaged Ct-value from three various reference genes (*Gusb*, *Hprt*, *Rn18s*) was used for calculation. The presented data are means ± SEM. *, **, *** *p* < 0.05, *p* < 0.01, and *p* < 0.001, respectively. *N* = 6 **(A–C)** and 7 **(D)**. Each data point (○) represents an individual animal. Statistical analyses were conducted using one-way ANOVA followed by the Bonferroni post hoc test, selected based on data distribution.

To assess the impact of PIC-induced MIA on amyloid-related processes in middle-aged mice, we quantified Aβ_1-40_ and Aβ_1-42_ levels using commercial ELISA assays. As presented in Figure 3A, Aβ_1-40_ showed a significant tendency to increase (*p* = 0.054) in the hippocampus. The levels of Aβ_1-42_ were not increased (Figure 3B). However, the total level of Aβ, calculated as a sum of Aβ_1-40_ and Aβ_1-42_, was significantly increased in the MIA group (Figure 3C). Also, the mRNA level of the *App* gene was increased in the hippocampus in the MIA group (Figure 3D). Fourteen days of treatment with OTX-015 significantly reduced Aβ levels in both the MIA-exposed group and in animals not subjected to prenatal inflammatory stress. However, OTX-015 did not impact the *App* gene expression in the hippocampus.

**Figure 4:**
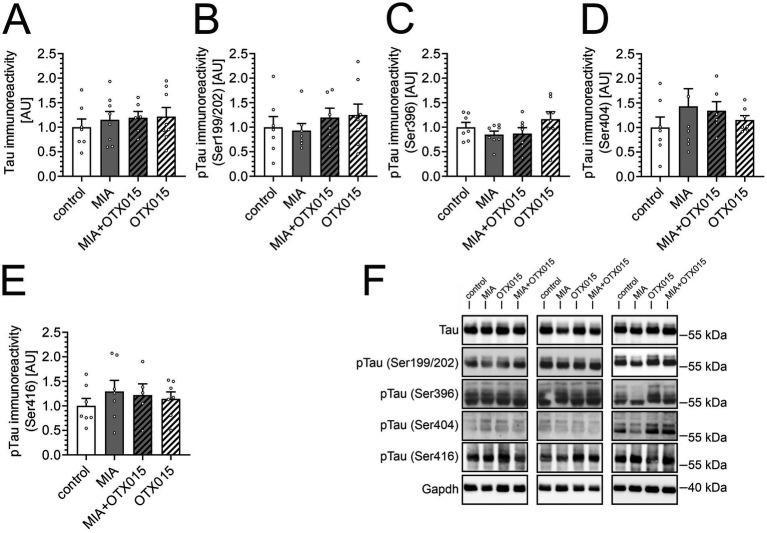
The effect of MIA and OTX-015 on Tau level and phosphorylation. PIC (20 mg/kg b.w.) was injected intraperitoneally at gestation day 17 to female mice. An inhibitor of BET proteins, OTX-015 (100 mg/kg b.w. daily), was administered orally to 12-month-old offspring males for 14 days (weeks 50–51), then animals were decapitated, and the brain tissue was collected. **(A–E)** The level and phosphorylation of Tau protein were analyzed in the hippocampus using Western blotting. Data were normalized on the immunoreactivity of Gapdh. The presented data are means ± SEM. *N* = 6–8 **(A,B)**, 7–8 **(C)**, 6–7 **(D)** and 5–7 **(E)**. Each data point (○) represents an individual animal. **(F)** Three representative sets of Western blot images are shown, derived from 12 individual animals. Statistical analyses were conducted using one-way ANOVA followed by the Bonferroni post hoc test, selected based on data distribution.

Increased Tau phosphorylation in the brain is a typical feature of AD, but alterations of phospho-Tau levels in the hippocampus were also observed during LPS-evoked systemic inflammation in mice (Czapski et al., 2016) and in adolescent rats after LPS-evoked MIA (Cieślik et al., 2020a). Therefore, in the next step, we performed Western blot analysis of the level and phosphorylation of Tau protein in our experimental conditions. The results demonstrated that MIA did not affect the total levels of Tau protein or phosphorylation (Figure 4). Treatment with OTX-015 also did not significantly impact the Tau protein in the investigated groups.

Because BET proteins are epigenetic regulators of gene expression, we analyzed the possible effect of OTX-015 on the levels of mRNA for several proteins related to amyloid-β metabolism in the hippocampus. We tested selected genes for secretases (alpha-secretase *Adam10*; beta-secretase *Bace1*; gamma-secretase *Psen1*, *Psen2*, *Aph1b*, *Ncstn*), and for enzymes responsible for degradation (*Mme*) and clearing (*Abca1*) of Aβ. As presented in Table 1, MIA evoked an increase in the level of *Bace1*, *Aph1b*, and *Mme* in the hippocampi of 12-month-old progeny. OTX-015 did not prevent MIA-induced changes; on the contrary, it exacerbated the increase in *Bace1* expression in MIA animals. Additionally, OTX-015 upregulated *Bace1* expression in animals that were not exposed to prenatal stress.

**Table 1:** The effect of MIA and OTX-015 on the expression of amyloidogenesis-related genes in the hippocampus of 12-month-old male mice.

	Control	MIA	MIA+OTX015	OTX015
	[RQ]	[RQ]	[RQ]	[RQ]
*Adam10*	1.00 ± 0.09 (7)	0.98 ± 0.12 (8)	1.14 ± 0.06 (7)	0.89 ± 0.16 (8)
*Bace1*	1.00 ± 0.13 (7)	1.45 ± 0.04 (7)**	1.89 ± 0.06 (6)##	1.63 ± 0.08 (6)***
*Psen1*	1.00 ± 0.20 (7)	0.94 ± 0.13 (8)	1.18 ± 0.11 (7)	1.04 ± 0.22 (8)
*Psen2*	1.00 ± 0.05 (7)	0.93 ± 0.06 (8)	1.25 ± 0.14 (7)	0.96 ± 0.07 (8)
*Aph1b*	1.00 ± 0.33 (7)	2.40 ± 0.25 (8)**	2.87 ± 0.17 (6)	1.27 ± 0.33 (8)
*Ncstn*	1.00 ± 0.22 (7)	1.61 ± 0.07 (8)	2.09 ± 0.31 (7)	1.81 ± 0.34 (8)
*Mme*	1.00 ± 0.34 (7)	2.71 ± 0.05 (7)***	2.49 ± 0.08 (6)	1.46 ± 0.38 (8)
*Abca1*	1.00 ± 0.07 (7)	1.05 ± 0.05 (8)	1.23 ± 0.04 (7)	1.19 ± 0.06 (8)

PIC (20 mg/kg b.w.) was injected intraperitoneally at gestation day 17 to female mice. An inhibitor of BET proteins, OTX-015 (100 mg/kg b.w. daily), was administered orally to 12-month-old offspring males for 14 days (weeks 50–51), then animals were decapitated, and the brain tissue was collected. The level of mRNA was measured by using qPCR and calculated by the ΔΔCt method. The presented values are mean ± SEM (*N*). **, *** *p* < 0.01, and *p* < 0.001, respectively, compared with the control group. ## *p* < 0.01, compared with the MIA group. RQ, relative quantification.

**Figure 5:**
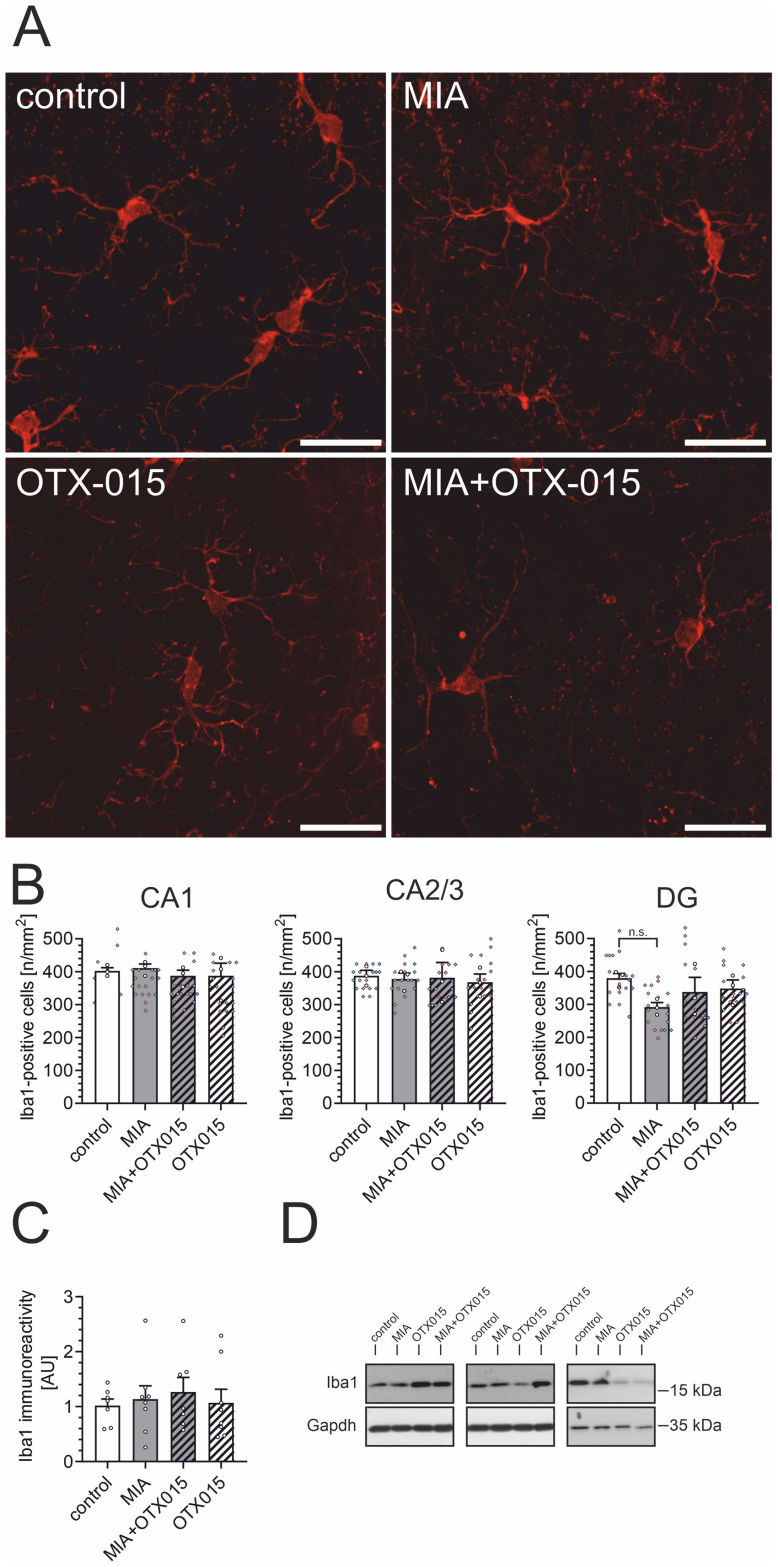
The effect of MIA and OTX-015 on microglial morphology and density in the hippocampus. PIC (20 mg/kg b.w.) was injected intraperitoneally at gestation day 17 to female mice. An inhibitor of BET proteins, OTX-015 (100 mg/kg b.w. daily), was administered orally to 12-month-old offspring males for 14 days (weeks 50–51), then animals were perfused and brain tissue slices were subjected to immunohistochemical analysis using anti-Iba1 antibodies. **(A)** Representative pictures illustrating Iba1-specific cells (microglia) in the CA1 region of the hippocampus were presented. Scale bar = 20 μm. **(B)** Semi-quantitative analysis of the number of visible Iba1-positive microglial cells per 1 mm^2^ in the CA1, CA2/3, and DG regions of the hippocampus. **(C)** The level of a marker of microglia (Iba1) was analyzed by Western blotting. Data were normalized on the immunoreactivity of Gapdh. **(D)** Three representative sets of Western blot images are shown, derived from 12 individual animals. *N* = 3 **(B)** and 7–8 **(C)**. Each data point (○) represents an individual animal. The counting results from the individual images that contribute to each animal’s score are shown as colored diamonds (◊), with each color representing a different animal. Statistical analyses were conducted using one-way ANOVA followed by the Bonferroni post hoc test, selected based on data distribution.

An alternative mechanism that may contribute to alterations in Aβ levels in the brain is the activity of microglial cells. Therefore, we performed an immunofluorescence analysis of Iba1-positive microglial cells in the hippocampal regions: CA1, CA2/3, and DG. As demonstrated in Figures 5A,B, neither MIA nor OTX-015 had any evident impact on the morphology or density of microglia. Then, we assessed the level of microglial marker Iba1 in the hippocampus. As shown in Figures 5C,D, the immunoreactivity of Iba1 was not affected by MIA or OTX-015, confirming that the number of microglia was not changed in our experimental conditions. Also, other markers of microglial activation, like CD68 or CD206, were not changed in our experimental conditions (data not shown).

**Figure 6:**
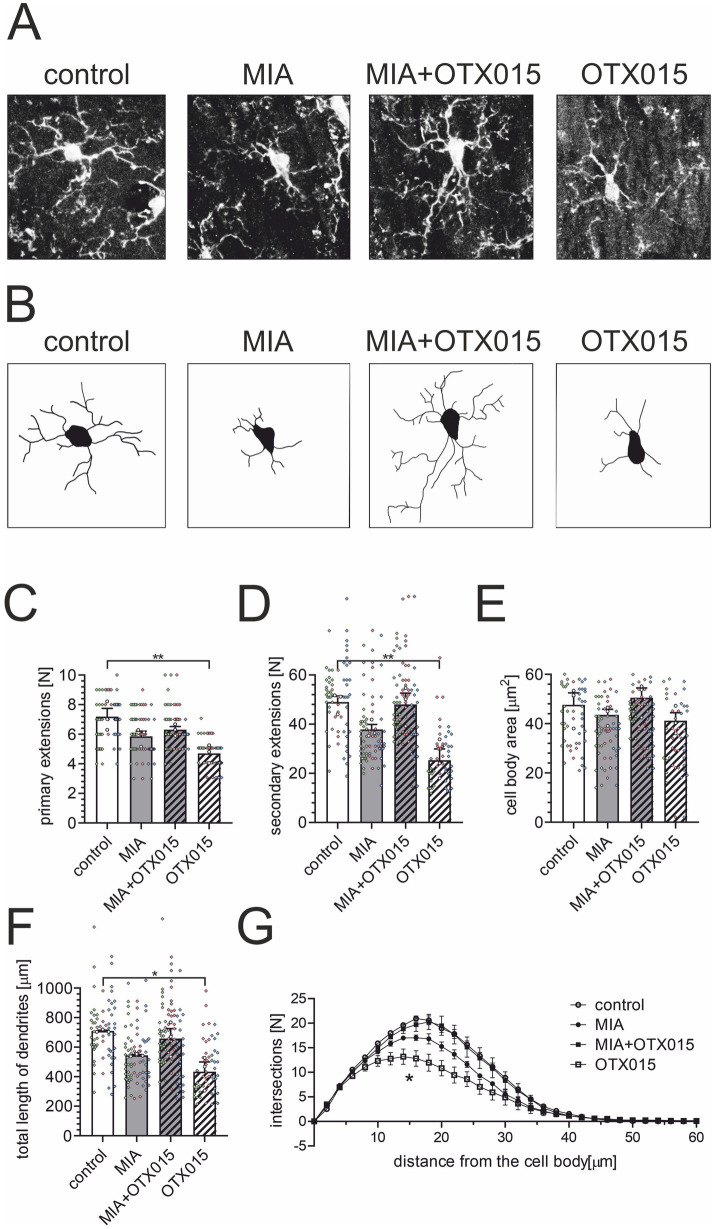
The effect of MIA and OTX-015 on glial cells in the hippocampus – a quantitative analysis. PIC (20 mg/kg b.w.) was injected intraperitoneally at gestation day 17 to female mice. An inhibitor of BET proteins, OTX-015 (100 mg/kg b.w. daily), was administered orally to 12-month-old offspring males for 14 days (weeks 50–51), then animals were euthanized, and the brain tissue was analyzed. The morphometric analysis of Iba1-positive (microglial) cells in the hippocampus was performed using the NeuronJ plugin for ImageJ Fiji software. The crucial steps in the morphology analysis was converting image to an 8-bit format **(A)** and processes tracing using the NeuronJ plugin for Fiji **(B)**. Representative images were shown. The morphometric analysis included the number of primary extensions **(C)**, the number of secondary extensions **(D)**, the cell body area **(E)**, and the total length of dendrites **(F)**. **(G)** Sholl analysis of the branching complexity of microglia in the hippocampus was performed with the SNT plugin for ImageJ Fiji. Data are presented as the mean value ± SEM. *, ** *p* < 0.05 and *p* < 0.01, respectively, compared with the control group. *N* = 3. Each data point (○) represents an individual animal. The results from the individual cells that contribute to each animal’s score are shown as colored diamonds (◊), with each color representing a different animal. Statistical analyses were conducted using one-way ANOVA followed by the Bonferroni post hoc test, selected based on data distribution.

Given that a simple estimation of microglial morphology in the hippocampus may not be sensitive enough to detect subtle changes, in the next step, we conducted a detailed quantitative analysis in the next step to further investigate microglial functionality in the CA1 field. CA1 pyramidal neurons are known to be highly vulnerable to Tau accumulation, mitochondrial impairment, and early synaptic degeneration—features that are paralleled by local microglial activation in multiple models of neurodegeneration (Gylys et al., 2004; Sisková et al., 2010). This region is also more sensitive to NMDA-induced excitotoxicity than CA3 or DG, and this susceptibility is abolished in microglia-depleted preparations, indicating a microglia-dependent component of regional vulnerability (Vinet et al., 2012). Based on these region-specific properties, CA1 was selected as a primary site for detecting microglial responses to OTX-015 in our MIA model. The morphometric software-supported (Babiec et al., 2023) analysis of Iba1-positive cells showed no significant differences in the number of primary and secondary extensions (Figures 6C,D), the area of cell bodies (Figure 6E), or the total length of extensions (Figure 6F) in the MIA group compared to the control group. Similarly, there were no changes in the arborization of microglia (Figure 6G) in animals exposed to MIA. However, there was a slight tendency for decreased microglial arborization in MIA animals, which was not observed in MIA animals treated with OTX-015 (Figures 6D–G). Surprisingly, in the control animals, treatment with OTX-015 resulted in a decrease in microglial arborization (Figures 6C,D,F,G).

**Figure 7:**
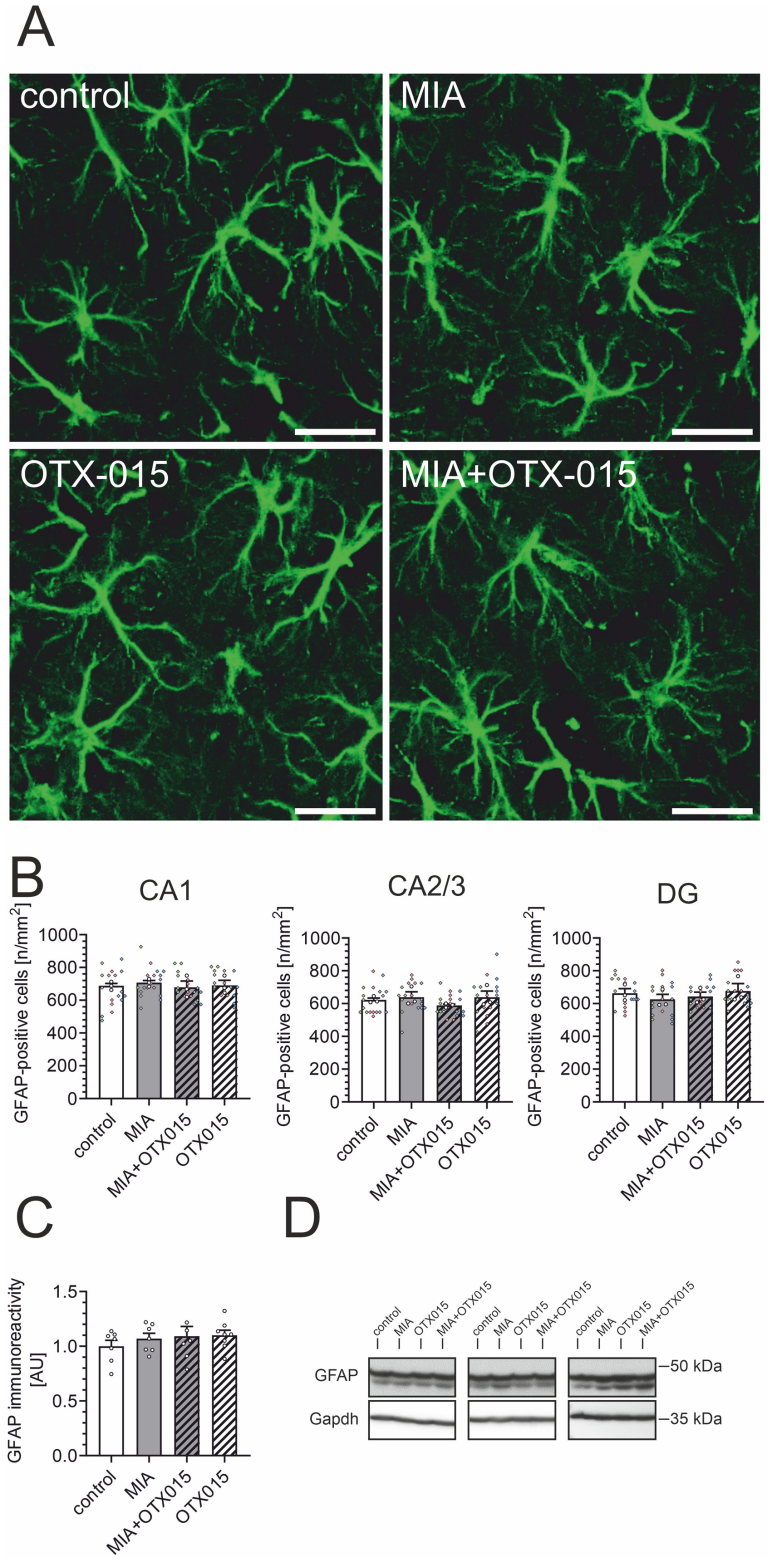
The effect of MIA and OTX-015 on astrocyte morphology and density in the hippocampus. PIC (20 mg/kg b.w.) was injected intraperitoneally at gestation day 17 to female mice. An inhibitor of BET proteins, OTX-015 (100 mg/kg b.w. daily), was administered orally to 12-month-old offspring males for 14 days (weeks 50–51), then animals were perfused and brain tissue slices were subjected to immunohistochemical analysis using anti-GFAP antibodies. **(A)** Representative pictures illustrating GFAP-specific cells (astrocytes) in the CA1 region of the hippocampus were presented. Scale bar = 20 μm. **(B)** Semi-quantitative analysis of the number of visible GFAP-positive cells per 1 mm^2^ in the CA1, CA2/3, and DG regions of the hippocampus. **(C)** The level of a marker of astrocytes (GFAP) was analyzed by Western blotting. Data were normalized on the immunoreactivity of Gapdh. **(D)** Three representative sets of Western blot images are shown, derived from 12 individual animals. *N* = 3 **(B)** and 7–8 **(C)**. Each data point (○) represents an individual animal. The counting results from the individual images that contribute to each animal’s score are shown as colored diamonds (◊), with each color representing a different animal. Statistical analyses were conducted using one-way ANOVA followed by the Bonferroni post hoc test, selected based on data distribution.

In the next step, we performed an immunofluorescence analysis of GFAP-positive astrocytes in the hippocampal regions: CA1, CA2/3, and DG. As demonstrated in Figures 7A,B, neither MIA nor OTX-015 had any evident impact on the morphology or density of astrocytes. We then assessed the level of astrocytic marker GFAP in the hippocampus. As shown in Figures 7C,D, the immunoreactivity of GFAP was not affected by MIA or OTX-015, confirming that the number of astrocytes remained unchanged under our experimental conditions.

Next, we utilized quantitative PCR, a highly sensitive technique for detecting subtle variations in mRNA levels of inflammation-related genes (Table 2). Our analysis indicated that MIA did not induce significant changes in the expression of either pro-inflammatory or anti-inflammatory genes. However, a trend toward increased expression of *Il6* and *Nos2* was observed. Notably, treatment with OTX-015 did not affect the expression of inflammation-related genes.

**Table 2:** The effect of MIA and OTX-015 on the expression of inflammation-related genes in the hippocampus of 12-month-old male mice.

	Control	MIA	MIA+OTX015	OTX015
	[RQ]	[RQ]	[RQ]	[RQ]
*Il1b*	1.00 ± 0.16 (7)	0.83 ± 0.03 (6)	0.89 ± 0.07 (7)	0.79 ± 0.09 (8)
*Il6*	1.00 ± 0.19 (7)	1.53 ± 0.12 (8) &	1.71 ± 0.23 (7)	1.35 ± 0.22 (8)
*Tnf*	1.00 ± 0.27 (7)	1.13 ± 0.13 (8)	0.79 ± 0.15 (6)	0.78 ± 0.16 (8)
*Nos2*	1.00 ± 0.08 (6)	1.50 ± 0.17 (8) &	1.39 ± 0.28 (7)	0.92 ± 0.18 (8)
*Arg1*	1.00 ± 0.08 (6)	0.98 ± 0.12 (8)	0.88 ± 0.07 (6)	0.89 ± 0.16 (8)

PIC (20 mg/kg b.w.) was injected intraperitoneally at gestation day 17 to female mice. An inhibitor of BET proteins, OTX-015 (100 mg/kg b.w. daily), was administered orally to 12-month-old offspring males for 14 days (weeks 50–51), then animals were decapitated, and the brain tissue was collected. The level of mRNA was measured by using qPCR and calculated by the ΔΔCt method. The presented values are mean ± SEM (*N*). & *p* < 0.05, compared with the control group using the Student t-test. RQ, relative quantification.

**Figure 8:**
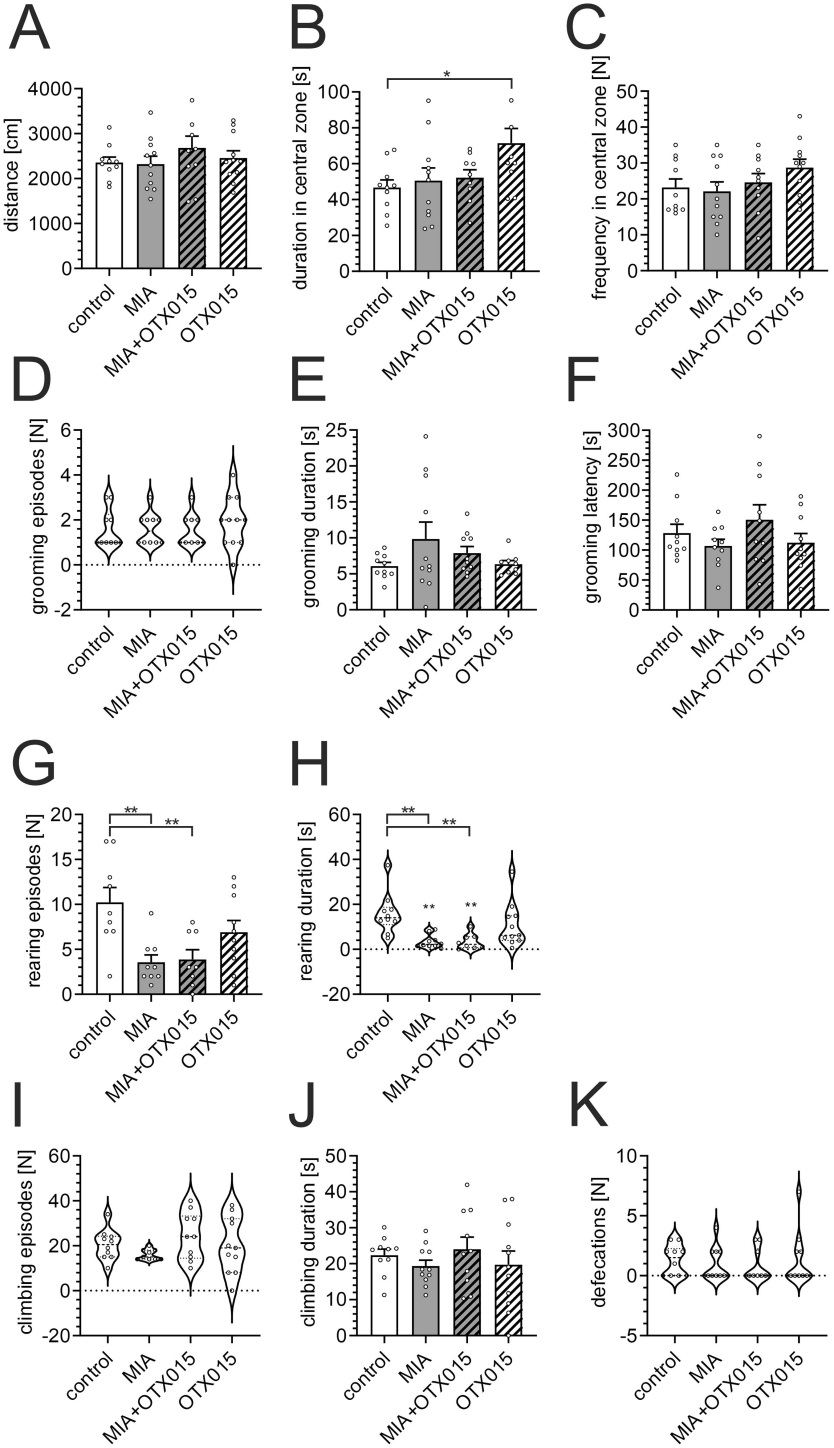
The effect of MIA and OTX-015 on exploratory activity and anxiety-related behavior in 12-month-old male mice. The exploratory activity and anxiety-related behavior of mice were analyzed in an open-field test: **(A)** the total distance traveled by animals, **(B)** the time spent in the central zone, **(C)** the number of entries to the central zone, **(D)** the number of self-grooming events, **(E)** the total time spent on self-grooming **(F)** the latency to the first self-grooming event, **(G)** the number of rearing events, **(H)** the duration of rearing events, **(I)** the number of climbing events, **(J)** the duration of climbing events, and **(K)** the number of defecations. Data **(A–C,E–G,J)** represent the mean values ± SEMs. Data not normally distributed **(D,H,I,K)** are presented as violin plots with all data points. *, ** *p* < 0.05 and *p* < 0.01, respectively, compared with the control group. *N* = 8–11. Each data point (○) represents an individual animal. Statistical analyses were conducted using either one-way ANOVA followed by the Bonferroni post hoc test, or the Kruskal–Wallis test followed by Dunn’s post hoc multiple comparisons test, as appropriate based on data distribution.

Finally, we measured the effect of MIA and OTX-015 on the behavior of middle-aged mice. We have investigated the exploratory activity and anxiety-related behaviors in the open-field test. As shown in Figure 8A, the total distance traveled during the test was not significantly affected in MIA-exposed or OTX-015-treated mice, indicating that neither mobility nor exploratory activity was altered under our experimental conditions. Rodents naturally avoid open areas, so changes in the frequency and duration of entries into the central zone of the open-field chamber are a measure of anxiety-related behavior. While MIA exposure did not affect central zone entries or the time spent in this area, non-MIA animals treated with OTX-015 significantly increased time spent in the central zone (Figures 8B,C).

Additionally, behaviors such as grooming, rearing, climbing, and defecation are commonly interpreted as markers of anxiety-related responses (Figures 8D–K). In our study, MIA exposure, regardless of OTX-015 treatment, specifically reduced the frequency and duration of rearing episodes, suggesting the presence of anxiety-related behavior in MIA-exposed animals.

**Figure 9:**
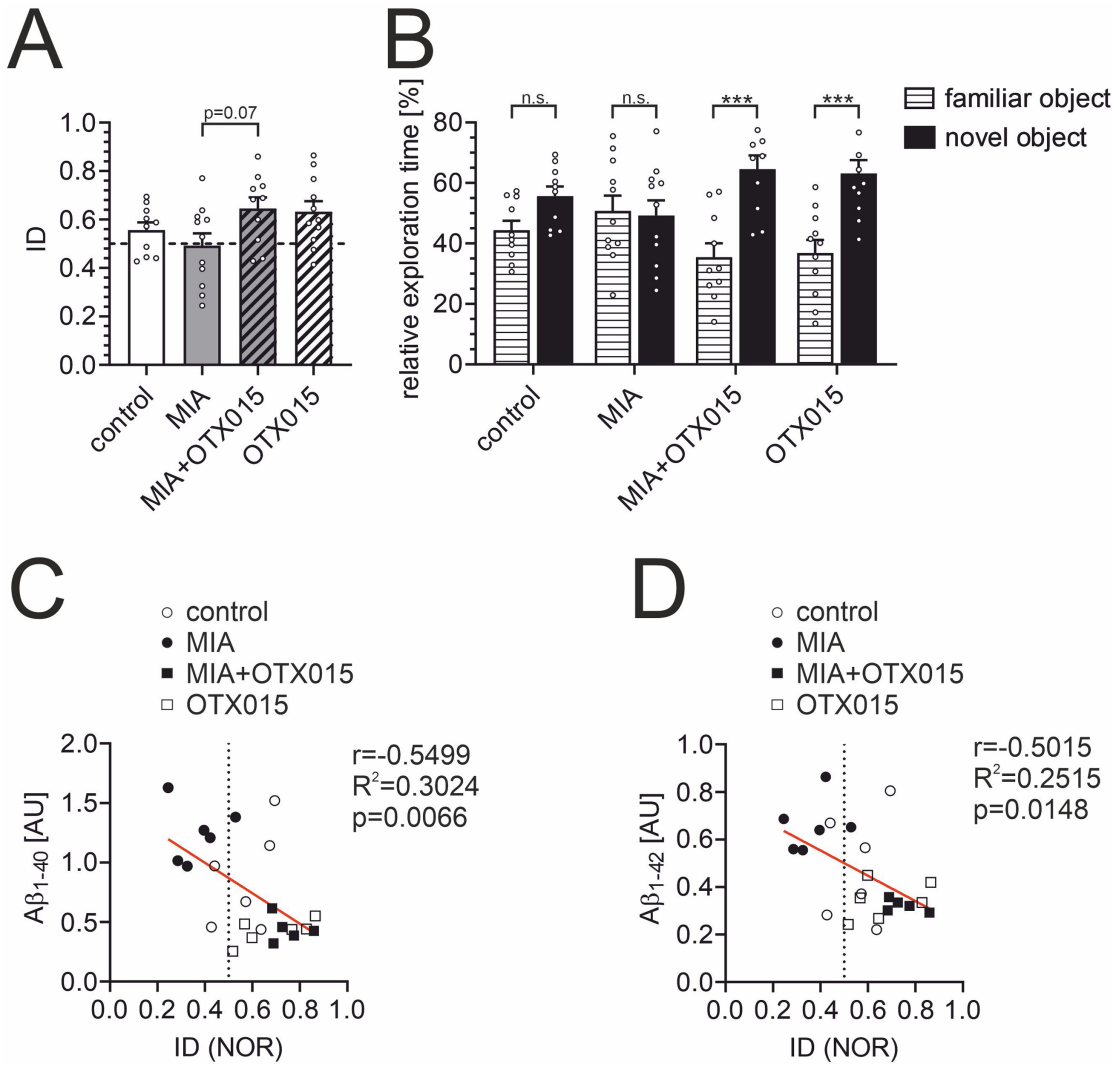
The effect of MIA and OTX-015 on memory function in 12-month-old male mice. The memory function of animals was analyzed in a novel object recognition (NOR) test. **(A)** The index of discrimination (ID) was calculated based on time spent exploring the novel object versus the familiar object, as described in the Methods section. **(B)** Relative exploration time of familiar vs. novel object in the NOR test. **(C)** The analysis of the correlation of the data from the NOR test with the levels of Aβ_1-40_ and Aβ_1-42_ **(D)** in corresponding animals was performed using Pearson’s correlation test. Presented results are means ± SEM from *N* = 10–11 animals in each group. n.s. – not significant, ***p < 0.001. Each data point (○) represents an individual animal. Statistical analyses were conducted using one-way ANOVA followed by the Bonferroni post hoc test, selected based on data distribution.

The novel object recognition (NOR) test is a widely utilized assay to evaluate animals’ memory. Animals that recall the objects presented during the initial test session prefer the novel object in the subsequent session. Therefore, the index of discrimination (ID) above 0.5 indicates that animals remember the objects, and an ID of about 0.5 shows that animals do not remember the objects. Our previous studies using the same NOR assay protocol observed that ID for young (2–3 month-old) adult male mice is above 0.6. As presented in Figure 9A, the ID for the control group is 0.56, indicating that 12-month-old animals have difficulty distinguishing between novel and familiar objects 120 min after the presentation session. This reduced ID may explain why the difference between the control and MIA groups (ID = 0.492) did not achieve statistical significance. We assume that a shorter delay between two sessions could give more conclusive results in aged animals. However, OTX-015 presented a strong tendency (*p* < 0.1) to improve cognitive function (Figure 9A). Moreover, the data were also presented as relative exploration time to display the difference between animals’ interest in familiar versus novel objects (Figure 9B). It is evident that animals treated with OTX-015, regardless of MIA exposure, spent significantly more time exploring the novel object than the familiar one (*p* < 0.001), indicating that they could effectively distinguish between them and retain the memory of the familiar object. In contrast, despite some tendency in the control group, the control and MIA groups spent similar amounts of time exploring novel and familiar objects. This suggests that these animals did not differentiate between the objects and thus failed to remember the familiar one. This result indicates the significant improvement in cognitive function in animals treated with OTX-015. Finally, we performed Pearson’s correlation analysis between ID (memory function) and Aβ levels in individual animals. The results presented in Figures 9C,D show a strong negative correlation.

## 4 Discussion

Alzheimer’s disease is characterized by the pathological accumulation of misfolded proteins in the brain, particularly Aβ and Tau. The aggregation of these proteins drives neurodegeneration, leading to neuronal loss and a consequent decline in the production of neurotransmitters essential for cognitive function. Moreover, the toxic forms of Aβ are known to activate glial cells, including microglia and astrocytes, which leads to chronic neuroinflammation. This inflammatory response contributes to synaptic loss and neuronal death, further exacerbating the cognitive decline associated with AD. Currently studied therapeutic strategies primarily target the mechanisms underlying the formation and clearance of Aβ and Tau, as well as the inflammatory response (Scheltens et al., 2021). The clinical efficacy of these treatments remains limited, underscoring the need for innovative therapeutic approaches (Passeri et al., 2022). The potential role of BET proteins in the progression of AD presents a promising avenue for exploration, although this area remains underinvestigated (Nikkar et al., 2022). In the current study, a mouse model of maternal immune activation (MIA) was utilized to investigate how prenatal immune challenges contribute to long-term alterations in Aβ levels, Tau phosphorylation, and neuroinflammation – crucial players in the pathomechanism of AD. This research model was selected based on the study by Krstic et al. (2012), which demonstrated that immune challenges during critical periods of prenatal brain development trigger neuroinflammation, promote amyloidogenesis and Tau-related pathology, resulting in learning and memory deficits in later stages of offspring life. These findings support the hypothesis that early-life immune events can increase susceptibility to neurodegenerative diseases in adulthood, warranting further research into early-life interventions. Furthermore, given emerging evidence that inhibiting BET proteins may alleviate cognitive deficits associated with AD and enhance neuroprotection, we examined the effects of BET protein modulation using OTX-015, a selective BET inhibitor (Eischer et al., 2023). The oral dose of OTX-015 was chosen based on prior literature and preclinical studies that demonstrated its efficacy and tolerability in animal models (Boi et al., 2015; Yu et al., 2022). OTX-015, similar to its analog JQ1, has been demonstrated to cross the blood–brain barrier, which makes it a valuable tool for pharmacologically targeting central nervous system pathologies (Matzuk et al., 2012; Berenguer-Daizé et al., 2016). However, it cannot be fully excluded that some peripheral effects could also contribute to its impact on the brain. The drug was administered orally to minimize animal distress, providing a more humane route of administration compared to intraperitoneal injection. After oral administration, OTX-015 penetrated the brain and demonstrated potent efficacy in a mouse glioblastoma model, offering increased survival and reduced tumor progression without significant toxicity (Berenguer-Daizé et al., 2016). This inhibitor has also been investigated in human trials for a different condition, suggesting that oral administration could be a viable therapeutic approach. These findings are consistent with previous studies, which indicate that the pharmacokinetics of the compound support oral delivery, potentially improving patient compliance and the feasibility of treatment (Dombret et al., 2014). To ensure efficient absorption and ease of administration, OTX-015 was dissolved in peanut butter, a lipophilic, palatable, and simple carrier, facilitating easy drug consumption by the mice. In compliance with ethical standards and to minimize animal suffering, we adhered to the 3Rs principle—Replacement, Reduction, and Refinement—throughout our experimental design. Specifically, the use of oral administration, a non-invasive method, reduced physical stress compared to more invasive procedures (MacArthur Clark, 2018; Azkona, 2023). Notably, we observed a significant increase in the mean duration of voluntary consumption of OTX-015 compared to peanut butter alone, which was consistent throughout the 14-day treatment period (Figure 1D). The reason for this decreasing propensity to voluntary drug intake is unknown. Information regarding the taste or smell of OTX-015 is unavailable. However, OTX-015 evoked distortion of the sense of taste in humans in a clinical phase 1 study (Amorim et al., 2016). Therefore, we cannot exclude the possibility that the attractiveness of peanut butter with OTX-015 could decrease over time. Also, this change in consumption behavior could suggest that BET inhibition may have unintended, off-target consequences that affect animals’ overall well-being. Specifically, such behavioral alterations might signal underlying discomfort or an altered metabolic state, which could be indicative of toxicity or adverse physiological responses. These findings underscore the importance of further investigation into the broader physiological effects of BET inhibition, particularly in the context of prolonged exposure. Changes in voluntary consumption patterns may serve as an early indicator of potential adverse effects that extend beyond the primary therapeutic objectives, such as cognitive enhancement or amyloid reduction. Given the critical role of safety in drug development, such behavioral changes must be carefully assessed to ensure that BET inhibitors, while offering therapeutic benefits, do not pose significant risks to overall health. Further toxicological studies are essential to refine the safety profile of BET inhibitors and ensure their viability as therapeutic agents for neurodegenerative diseases, such as AD.

Our quantitative PCR analysis revealed a significant upregulation of *Brd4* mRNA in the hippocampus of the MIA group, while *Brd2* and *Brd3* mRNA levels remained unchanged (Figure 2). In contrast, ELISA assays failed to show a corresponding increase in Brd4 protein levels, suggesting a divergence between mRNA and protein expression. This discrepancy may reflect the inherent limitations of these methodologies, as differences in assay sensitivity and specificity are well-established. Such variations emphasize the complexity of post-transcriptional regulation and underscore the challenges in interpreting mRNA and protein expression data, particularly when utilizing distinct analytical approaches (Savino et al., 2009; Eischeid et al., 2021). Remarkably, the selective upregulation of Brd4 was also observed in our previous *in vitro* (Matuszewska et al., 2025) and *in vivo* experiments (data not shown, submitted for publication) using lipopolysaccharide to trigger an inflammatory response.

Our most interesting finding is the observation of increased Aβ levels in the hippocampi of animals prenatally exposed to inflammatory challenge (Figure 3). Moreover, BET inhibition significantly reduced Aβ levels in both control animals and those exposed to MIA. Importantly, this observation is further supported by the results of our experiments on adult mice, where we observed an attenuation of Aβ levels in the brain after using JQ1, an analog of OTX-015 (data not shown, submitted for publication). Neuroinflammation, such as that induced in the MIA model, is known to activate several transcription factors, including NF-κB, which plays a prominent role in the pathomechanism of AD (Sun E. et al., 2022a). The *App* gene is a target gene of NF-κB, therefore, neuroinflammation could upregulate Aβ levels simply by enhancing *App* expression. However, although BET protein inhibitors are known to attenuate the expression of NF-κB-controlled genes (Huang et al., 2017), in our experimental conditions, they did not impact the expression of *Il1b*, *Il6*, *Nos2*, *and Tnf,* which are NF-κB-controlled genes (Table 2). This suggests that other, NF-κB-independent, mechanisms play a primary role in the anti-amyloid effect of OTX-015. Significantly, the promoter of the gene encoding BACE1, the aspartyl protease which is directly responsible for Aβ generation, harbors a binding site for NF-κB (Rossner et al., 2006). In accordance with the above interpretation, OTX-015 did not prevent MIA-evoked increase in *Bace1* expression. On the contrary, BET inhibition enhanced *Bace1* transcription, which is consistent with previous observations (Zhang et al., 2022). Further investigation into the intricate relationship between BET inhibition, neuroinflammation, and *App* regulation is needed to elucidate the specific molecular mechanisms underlying these observations. Such studies will help clarify the broader implications of BET inhibition in neurodegenerative conditions and its potential as a therapeutic strategy for diseases associated with Aβ accumulation. The possible mechanism of anti-amyloid action of OTX-015, which is worth analysis in future studies, is activating autophagy. This effect can be achieved through the modulation of signaling pathways by BET inhibitors, which indirectly promote autophagy activation (Jaeger et al., 2010; Cummings et al., 2024; Zeng et al., 2024). Prior studies have shown that BET inhibition can induce autophagy by modulating the AMPK-mTOR-ULK1 signaling cascade (Korb et al., 2015; Jang et al., 2017; Li et al., 2020). Given the role of autophagy dysregulation in AD, stimulating this cellular process has emerged as a promising therapeutic strategy for removing and clearing Aβ deposits (Friedman et al., 2015; Rahman et al., 2020). Experimental data support the efficacy of numerous autophagy activators in reducing Aβ levels (Ordóñez-Gutiérrez et al., 2018; Pierzynowska et al., 2019; Kong et al., 2020). In summary, the literature suggests that such a cascade is feasible and may present a novel therapeutic strategy for AD. However, further clinical studies are essential to confirm the efficacy and safety of this approach in humans.

In parallel, the changes in Aβ levels were correlated with cognitive functions (Figure 9). Increased levels of Aβ have been consistently correlated with cognitive dysfunction, particularly in neurodegenerative diseases like AD (Näslund et al., 2000; Baker et al., 2017). Studies have shown that Aβ accumulation can directly impair cognitive functions by disrupting synaptic plasticity and increasing neuroinflammation (Fang et al., 2010; Ripoli et al., 2014; Rolland et al., 2020). Furthermore, emerging evidence highlights the role of peripheral clearance mechanisms, such as lymphatic drainage, in regulating Aβ levels and, consequently, cognitive performance. The dysfunction of these pathways can exacerbate cognitive decline, suggesting that both central and peripheral processes contribute to memory deficits in AD (Cheng et al., 2020). Additionally, therapeutic interventions targeting Aβ, such as anti-Aβ monoclonal antibodies, have shown that decreases in brain Aβ levels relate to slower cognitive decline (van Dyck et al., 2023). Furthermore, the relationship between changes in Aβ levels and cognitive function is central to understanding disease progression in AD (Abanto et al., 2024). These findings align with a growing body of literature suggesting that BET protein inhibition enhances cognitive functions and memory in AD contexts. BET protein inhibitor, JQ1, improved brain plasticity in wild-type and APP-expressing mice and rescued hippocampal-dependent cognitive deficits in a murine model of frontotemporal dementia (Benito et al., 2017; Quezada et al., 2021). Also, chronic administration of JQ1 significantly improved cognition deficits in rat models of AD (Badrikoohi et al., 2022; Nikkar et al., 2022). However, in 7-month-old 3 × Tg mice, JQ1 did not ameliorate learning and memory deficits (Magistri et al., 2016). On the other hand, prolonged administration of JQ1 evoked memory deficits in mice (Korb et al., 2015). Interestingly, the administration of JQ1 to young rats induced cognitive impairment in adult rats (Bilecki et al., 2021). It is noteworthy that Apabetalone (RVX-208), a distinct BET inhibitor, positively impacted cognitive performance in a randomized controlled trial conducted on a population of patients aged 70 years and older (Cummings et al., 2021). This convergence of results across studies reinforces the hypothesis that BET modulation could serve as a viable therapeutic strategy for AD. However, it is worth noting that although Aβ accumulation is associated with an increased risk and severity of cognitive decline, it is not the sole determinant. Aβ deposits do not necessarily lead to cognitive dysfunction in all individuals, and conversely, cognitive impairment can occur in the absence of significant amyloid pathology (Armstrong, 2014; Morris et al., 2014; Kepp et al., 2023). Therefore, given the complexity and diverse clinical manifestations of AD in the population (Korczyn and Grinberg, 2024), reducing Aβ alone may not be sufficient to restore cognitive function in all patients.

Tau expression and pathological phosphorylation are of particular interest in the conditions of the prenatal immune challenge and AD. In a rat model, MIA evoked by LPS-triggered neuroinflammation and microglial activation in adolescent offspring led to disrupted normal Tau function, and reduced Tau phosphorylation at Ser199/202 and Ser396, while phosphorylation at Ser416 remained unaffected (Cieślik et al., 2020b). The Knuesel group studied Tau protein levels and phosphorylation across aging in C57BL/6 J mice after PIC-induced MIA (Krstic et al., 2012). Their observations were inconclusive, as they presented age-dependent changes in phosphorylation at threonine 205 (Thr205), which increased at 6 and 15 months but decreased at 12 months. Our results (Figure 4) demonstrated that Tau level and phosphorylation were not changed in middle-aged MIA offspring. Also, OTX-015 did not affect Tau phosphorylation.

In MIA models, prenatal immune stress results in microglial hypertrophy, as evidenced by increased cell body size and decreased arborization in neonatal and young offspring (Loayza et al., 2023). These morphological changes reflect a shift towards a more activated microglial phenotype, often associated with impaired surveillance and synaptic pruning, particularly in hippocampal regions such as the dentate gyrus and CA1 area (Delorme et al., 2023; Green and Rowe, 2024). The reduced complexity of microglial processes following MIA may disrupt normal neurogenesis and neuronal connectivity, contributing to neurodevelopmental abnormalities. Our study did not observe evidence of microglial hypertrophy in MIA-exposed middle-aged offspring (Figures 5, 6). Surprisingly, OTX-015 affected microglial morphology in MIA-not-exposed animals by inducing process retraction. This phenotype may reflect a slightly heightened inflammatory response, which could alter microglial functions. Because we did not observe any anti-inflammatory effect of OTX-015 in our conditions, we speculate that the detected effect could result from some compensatory mechanisms after prolonged OTX-015 treatment. However, further studies are necessary to elucidate the precise mechanisms through which these alterations occur and influence hippocampal function.

While our study leverages the MIA model to investigate the epigenetic ramifications of maternal immune activation on offspring, it is essential to acknowledge the limitations of our results. Our study utilizes a mouse model, which may not fully recapitulate the complexities of human disease. The MIA model, thanks to which we observed molecular changes in the offspring of a pregnant female whose immune system was activated, is well known and described (Krstic et al., 2012; Garay et al., 2013). However, as previously suggested, inflammatory responses in mouse models often exhibit limited concordance with human disease, and the underlying regulatory pathways may diverge substantially (Seok et al., 2013; Diehl and Boyle, 2018). However, other studies demonstrated significant similarities in some mechanisms of response to inflammation between humans and mice (Shay et al., 2013; Takao and Miyakawa, 2015). Also, this study utilized only male animals to mitigate the potential influence of the estrous cycle on experimental outcomes. However, given the higher prevalence of AD in females, future studies should incorporate both sexes to ensure a more comprehensive understanding of the pathomechanism of the disease. Another limitation of our study is the sample size in immunohistochemical analysis. The number of animals is sufficient to obtain reliable results. However, only repeating the tests and increasing the study group could bring us closer to testing potential drugs and planning clinical trials (Bogue et al., 2023). Other important question, we did not answer, is how stable are OTX-015 evoked changes. To date, little is known about the durability of epigenetic modulation following BET inhibition in the context of MIA. While OTX-015 has been shown to efficiently alter chromatin accessibility and transcriptional activity in various disease models, the stability of these changes following drug withdrawal remains to be elucidated. Moreover, given the systemic nature of OTX-015’s effects and the complex interplay between immune activation, chromatin remodeling, and neurodevelopmental timing, long-term follow-up studies would be essential to clarify whether any MIA-induced alterations reappear or remain suppressed after treatment ends. Finally, we were unable to determine the specific molecular mechanism by which OTX-015 exhibits its anti-amyloid effect. Further research should explore alternative potential mechanisms.

Our results showed that orally administered BET protein inhibitor, OTX-015, reduced Aβ levels in the hippocampus and improved memory in mice. Further validation of experiments using OTX-015 for long periods and in different age frames is needed to determine the exact mechanism of the effects of BET inhibition. In conclusion, our findings support the further exploration of BET family protein inhibitors as a promising therapeutic strategy for Alzheimer’s disease. Expanding the repertoire of tested inhibitors and conducting studies on human tissues will be critical steps toward validating these findings. The prospect of initiating clinical trials based on our results could pave the way for novel interventions to improve memory and cognitive function in individuals afflicted with AD. Longitudinal studies with OTX-015 across diverse age groups will also be pivotal in assessing its long-term effects on cognition and behavior in preclinical and clinical settings.

## Data Availability

The original contributions presented in the study are included in the article/supplementary material, further inquiries can be directed to the corresponding author.
